# The Epidermal Cell Structure of the Secondary Pollen Presenter in *Vangueria infausta* (Rubiaceae: Vanguerieae) Suggests a Functional Association with Protruding Onci in Pollen Grains

**DOI:** 10.1371/journal.pone.0096405

**Published:** 2014-05-07

**Authors:** Patricia M. Tilney, Abraham E. van Wyk, Chris F. van der Merwe

**Affiliations:** 1 Department of Botany and Plant Biotechnology, University of Johannesburg, Auckland Park, Johannesburg, South Africa; 2 Department of Plant Science, University of Pretoria, Pretoria, South Africa; Institute of Botany, Chinese Academy of Sciences, China

## Abstract

Secondary pollen presentation is a well-known phenomenon in the Rubiaceae with particularly conspicuous pollen presenters occurring in the tribe Vanguerieae. These knob-like structures are formed by a modification of the upper portion of the style and stigma, together known as the stylar head complex. In the flower bud and shortly before anthesis, the anthers surrounding the stylar head complex dehisce and release pollen grains which adhere to the pollen presenter. The epidermal cells of the pollen presenter facing the anthers are radially elongated with a characteristic wall thickening encircling the anticlinal walls of each cell towards the distal end. These cells were studied in the pollen presenter of *Vangueria infausta* using electron and light microscopy in conjunction with histochemical tests and immunohistochemical methods. Other prominent thickenings of the cell wall were also observed on the distal and proximal walls. All these thickenings were found to be rich in pectin and possibly xyloglucan. The terms “thickenings of Igersheim” and “bands of Igersheim” are proposed to refer, respectively, to these wall structures in general and those encircling the anticlinal walls of each cell near the distal end. The epidermal cells have an intricate ultrastructure with an abundance of organelles, including smooth and rough endoplasmic reticulum, Golgi apparatus, mitochondria and secretory vesicles. This indicates that these cells are likely to have an active physiological role. The pollen grains possess prominent protruding onci and observations were made on their structure and development. Walls of the protruding onci are also rich in pectin. Pectins are hydrophilic and known to be involved in the dehydration and rehydration of pollen grains. We hypothesise that the thickenings of Igersheim, as well as the protruding onci of the pollen grains, are functionally associated and part of the adaptive syndrome of secondary pollen presentation, at least in the Vanguerieae.

## Introduction

**Figure 1:**
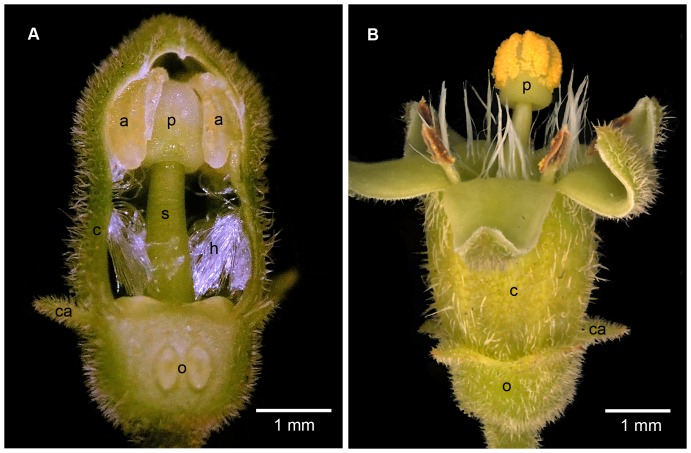
Macromorphology of secondary pollen presenter in closed and open flower. (A) Stereomicrograph of flower bud with the inferior ovary (o) and corolla (c) with some attached stamens cut away longitudinally to show the still-closed, dorsifixed anthers (a) at the same level as, and closely appressed to, the surface of the secondary pollen presenter (p) which terminates the style (s). The stigmatic surface is initially covered by the upper sterile portion of the anthers. The calyx lobes (ca) are relatively short. A prominent fringe of mainly downwardly directed hairs (h) lines the corolla tube. (B) Stereomicrograph of flower shortly after anthesis to show the spreading corolla lobes and pollen presenter carrying the pollen (yellow) above the rest of the flower (labels as in A). Shortly before anthesis, while still in bud, the anthers dehisce introrsely to release the pollen which adheres particularly to shallow longitudinal grooves in the pollen presenter. Some of the fringing hairs are directed upwards and protrude from the mouth of the corolla tube.

Secondary pollen presentation is a widespread phenomenon in the flowers of Rubiaceae and a synapomorphy for the tribe Vanguerieae [1]–[3]. In members of this tribe the upper portion of the style is modified to form a knob-like pollen presenter (“receptaculum pollinus”), tipped by the stigmatic surface, together forming a structural unit known as the stylar head complex [4]. In flower buds, the anthers surround and are appressed against the stylar head complex (Figure 1A). Just before anthesis, the anthers dehisce introrsely and release pollen which adheres to the pollen presenter. At anthesis (Figure 1B) the pollen presenter with adherent pollen grains is exposed, thereby presenting pollen to potential pollinators; the anthers then shrivel and become obsolete. Functionally the secondary pollen presenter has hitherto usually been viewed as a structure merely providing physical support for the adherent pollen grains.

In the Vanguerieae, the portions of the pollen presenter facing the anthers have markedly radially elongated epidermal cells with characteristic anticlinal wall thickenings towards the distal end (e.g. [4], [5]). The first report of these ring-like thickenings was by Igersheim ([4], p. 188), based on light microscopic studies of pollen presenters in various Vanguerieae. Igersheim ([4], p. 188) speculated that “they might represent, in addition to the strongly cutinized outer tangential walls of the epidermal cells, a second ‘mechanical barrier’ which prevents self-pollination (i.e., growth of the pollen deposited on the outside of the ‘stylar head’ into the interior).”

Pollen grains of several members of the Rubiaceae have apertures with prominent intine projections, known as protruding onci or pollen buds (e.g. [6]–[8]). Uncertainty still exists as to the distinction, if any, between these two structures [9]. Pollen buds are purported to be larger structures, containing a well-developed vacuole, which separate from the grain before the pollen is shed whereas protruding onci may or may not have cytoplasmic inclusions [9]. In a study of the protruding onci in the pollen of *Uncaria hirsuta* (Rubiaceae, tribe Naucleeae), Kuang et al. [9] observed differences in prominence and appearance in undehisced and dehisced anthers. However, these authors did not mention that *U. hirsuta* also displays secondary pollen presentation. Kuang et al. [9] also reported that the time of oncus formation in the course of pollen grain development depended on the taxon. To the best of our knowledge, no function has been suggested to date for protruding onci. Conventional internal onci associated with apertures in pollen grains (sometimes referred to as “Zwischenkörpen” in grasses) are rich in pectin. Onci play an important role during cycles of dehydration and rehydration of the pollen grains and also facilitate the emergence of the pollen tube during pollen germination [10]–[12].

*Vangueria infausta* Burch. subsp. *infausta* (wild-medlar), henceforth referred to as *V. infausta*, is a representative member of the Vanguerieae. It is a deciduous shrub or small tree native to eastern, south-central and southern Africa, and is mainly confined to sunny areas in wooded grassland and savannah, often in rocky places [13], [14]. The flowers are produced in early spring (September and October), usually before or together with the appearance of the new leaves. These are relatively small (corolla tube 3–4.5 mm long; lobes 3–4 mm long), greenish white and borne in large numbers in axillary clusters [13], [14]. The flowering period is brief, with flowers maturing sequentially over about two weeks, the individual flowers rarely lasting more than two days following anthesis. The flowers produce nectar and are visited by a diversity of insects, including honey bees (*Apis mellifera*), which are probably the main pollinator (own observations).

In an earlier unpublished study by us of the pollen presenters of various members of the Rubiaceae (poster, International Botanical Congress, Melbourne 2012), anticlinal epidermal cell wall thickenings of the type first reported by Igersheim [4] were noted in several taxa including in *V. infausta*. What is the composition of these wall thickenings? Might a better understanding of their composition and more information on the structure of the rest of the stylar head complex shed more light on the function of these thickenings? Material of *V. infausta* is readily available and so was selected for detailed investigation aimed at answering the above questions and at enhancing our knowledge of the anatomical aspects of secondary pollen presentation in the Vanguerieae in general. In the present contribution we report on the structure, particularly of the epidermal cells, of the secondary pollen presenter of *V. infausta* using light microscopy in conjunction with histochemical tests and immunohistochemistry, as well as scanning and transmission electron microscopy. Observations were also made on the protruding onci of the pollen grains during their development. Information here reported is used to assess the merits of the prevailing hypothesis [4] that the anticlinal wall thickenings of the epidermal cells of the pollen presenter may act as mechanical barriers preventing selfing. Based on structural evidence, we propose, for the first time, a possible functional association between protruding onci and secondary pollen presentation.

## Materials and Methods

### Ethics statement

Permission to collect material of the plant *Vangueria infausta* in the Melville Koppies Nature Reserve (26°10′11.19"S 27°59′16.81"E), Johannesburg, was granted by Wendy Carstens, Chairperson of the Melville Koppies Management Committee. Permission to collect material of this species in the Manie van der Schijff Botanical Garden (25°45′8.43"S 28°13′14.96"E), University of Pretoria, Pretoria, was obtained from Jason Sampson, Curator of the garden. *V. infausta* is a common and widespread species not protected under specific legislation.

### Plant material

The fresh material (flower buds at different stages of development and mature flowers) of *Vangueria infausta* was collected in the field at Melville Koppies Nature Reserve, Johannesburg (J) on 5 October 2010 (*Tilney s.n*. in JRAU) and in the Manie van der Schijff Botanical Garden, University of Pretoria, Pretoria (P) on 14 October 2012 (*Van der Merwe s.n*. in PRU). Herbarium material (*Stanton 7* and *Hemm 702*, in PRU) was used for some of the scanning electron microscope studies.

### Embedding methods, sectioning and viewing

#### 1. Glycol methacrylate embedding

Fresh material collected in J was fixed and preserved in FAA [formaldehyde (40%): glacial acetic acid: ethanol (96%): distilled water, 2:1∶10∶7]. The pollen presenters were separated from the rest of the mature flowers and, together with whole buds, were embedded in glycol methacrylate (GMA) [15]. Transverse and, for some stages of bud development, also longitudinal sections, 3–5 µm thick, were cut with a Porter-Blum Sorvall MT-1, U.S.A. ultramicrotome and placed on slides. Most of these slides were used to make permanent preparations using the periodic acid Schiff (PAS)/toluidine blue O staining method [15] and mounted with Entellan (Product 7961, E. Merck, Darmstadt) but some were left unstained and unmounted for the histological tests, comprising fluorescence microscopy (with auramine O) and immunohistochemical studies (see below). Samples were viewed with either a Nikon Optiphod light microscope (LM) fitted with a Nikon DXM 1200 digital camera or an Olympus CX-41camera and ColorView Soft Imaging System.

#### 2. Wax embedding

Samples fixed in 10% formalin were dehydrated with an ethanol series (30%, 50%, 70%, 90% and three changes of 100%) and placed in *tert*-butanol changed three times in 24 h. Wax pellets were added and left at room temperature to dissolve in the *tert*-butanol for 2 days. The vials with samples were then transferred to an oven at 60°C. The wax was replaced four times over a 2 day period after which samples were embedded in paraffin wax. Sections were cut 5 µm thick and placed on glass slides. The wax was removed with *tert*-butanol before use. Unstained sections were used for immunohistochemistry and high resolution scanning electron microscopy.

### Histological tests (using GMA-embedded unstained sections)

1. For the presence of de-esterified pectins: A 0.02% aqueous ruthenium red solution was added [16]. Positive results are indicated by red or pink colours.
2. For the presence of calcium pectate: 1% aqueous tannic acid and 3% aqueous ferric chloride solutions were used [16]. Black or blue-black colours indicate positive results.
3. For the presence of starch or xyloglucan: A solution of iodine dissolved in potassium iodide (IKI) was used for this test [17], [18]. A black or blue-black colour shows positive results.

### Immunohistochemical tests

Samples embedded in GMA and wax were used for fluorescence microscopy. For blocking non-specific binding sites, glass slides with adherent sections were incubated with 3% (w/v) fat free milk protein in phosphate-buffered saline (MP/PBS) in a moist chamber for 60 min. This was followed by incubation in five-fold diluted primary rat monoclonal antibodies in MP/PBS for 2 h at room temperature. Primary antibodies used (hybridoma cell culture supernatants) were LM19 [19] against de-esterified pectic homogalacturonan and LM20 [19] against methyl-esterified pectic homogalacturonan (Plantprobes, Leeds, UK). After three washes in PBS (at least 10 min for each change), sections were incubated with green-fluorescent Alexa Fluor 488 goat anti-rat IgG (H+L) (Invitrogen A-11006, Life Technologies, Johannesburg) antibodies diluted 100-fold in MP/PBS for 1 h in the dark at room temperature. Following antibody treatment, all sections were washed three times in PBS (at least 10 min for each change) and mounted in VECTASHIELD (Vector H-1000, Biocom Biotech, Centurion) antifade medium. Coverslips were sealed with nail polish around the edges. Fluorescence imaging was performed using a CLSM as for below.

### Fluorescence microscopy

Confocal fluorescence microscopy with the stain auramine O (Sigma-Aldrich 861030, Johannesburg; 0.01% w/v in 0.05 M Tris/HCl, pH 7.2) was used to study the cuticle/cutin. Sections of GMA-embedded material (see above) were placed in the stain for 15 min, rinsed with distilled water [20], [21] and then dried before mounting in immersion oil and sealing with nail varnish. Confocal laser imaging was performed using a confocal laser scanning microscope (CLSM) (Model LSM 510, ZEISS, Germany). Auramine O was excited using a 460 nm argon laser, and emission was collected at 550 nm. Samples embedded in GMA and wax were used for fluorescence microscopy as described in the immunohistochemical tests above.

### Scanning electron microscopy

Pollen presenters from buds and open flowers of herbarium specimens were removed and mounted on double-sided carbon tape, coated with gold and viewed with a JEOL 5800 LV scanning electron microscope (SEM). In addition, fresh samples of pollen presenters collected at P were fixed with a glutaraldehyde/formalin mixture, dehydrated with ethanol, critical point dried using liquid carbon dioxide (BioRad E3000 critical point drier, Polaron, West Sussex, UK) and mounted and viewed as above. Some of the latter samples were coated with carbon before viewing with a Zeiss Ultra FEG SEM.

### Transmission electron microscopy and immunogold labelling

Freshly collected pollen presenters from buds of varying ages as well as of mature flowers of *V. infausta* collected in P, were fixed in either 10% formalin (freshly prepared from paraformaldehyde) in 0.075 M sodium phosphate buffer or in a mixture of 2.5% glutaraldehyde and 2.5% formalin in the same buffer as above. Samples fixed in the 10% formalin were embedded in LR White while the glutaraldehyde/formalin-fixed samples were postfixed in 1% aqueous osmium tetroxide and embedded in SPI 812, a derivative of Epon 812. The whole bud was used in the case of the youngest specimen. Monitor transverse sections, 0.5 µm thick and stained with 0.2% toluidine blue dissolved in 0.5% sodium carbonate, were made for light microscopic viewing before ultrathin sections were cut to ensure that the latter were cut in the correct position and to facilitate their interpretation using TEM. Ultrathin transverse sections, about 60 nm thick, were cut using a Reichert-Jung Ultracut E microtome and examined with a JEOL JEM 2100F transmission electron microscope (TEM).

Antibody immunogold labelling for TEM was performed on sections of different developmental stages obtained from fresh material (collected in P) fixed in 10% formalin, embedded in LR White and mounted on gold grids. To prevent non-specific binding, the grid was floated (section side down) on a droplet (at least 20 µl) of 3% (w/v) bovine serum albumin in phosphate-buffered saline (BSA/PBS) on pink dental wax sheets in a moist chamber for 90 min. Thereafter the grid was transferred to a droplet of either one of two monoclonal primary (MP) antibodies (the same as for fluorescence microscopy; see above) diluted 5-fold and 100-fold in BSA/PBS and incubated for 90 min at room temperature. After three washes in PBS (at least 10 min for each change), grids were incubated with goat anti-rat IgG antibodies conjugated to 10 nm colloidal gold (Sigma-Aldrich G7035, Johannesburg) diluted 100-fold in MP/PBS for 1 h in the dark at room temperature. Following antibody treatment, grids were washed three times in PBS (at least 10 min for each change) and then extensively in distilled water. Grids were allowed to dry before being examined unstained in a JEOL JEM 2100F TEM.

## Results and Discussion

### Morphology of the stylar head complex

**Figure 2:**
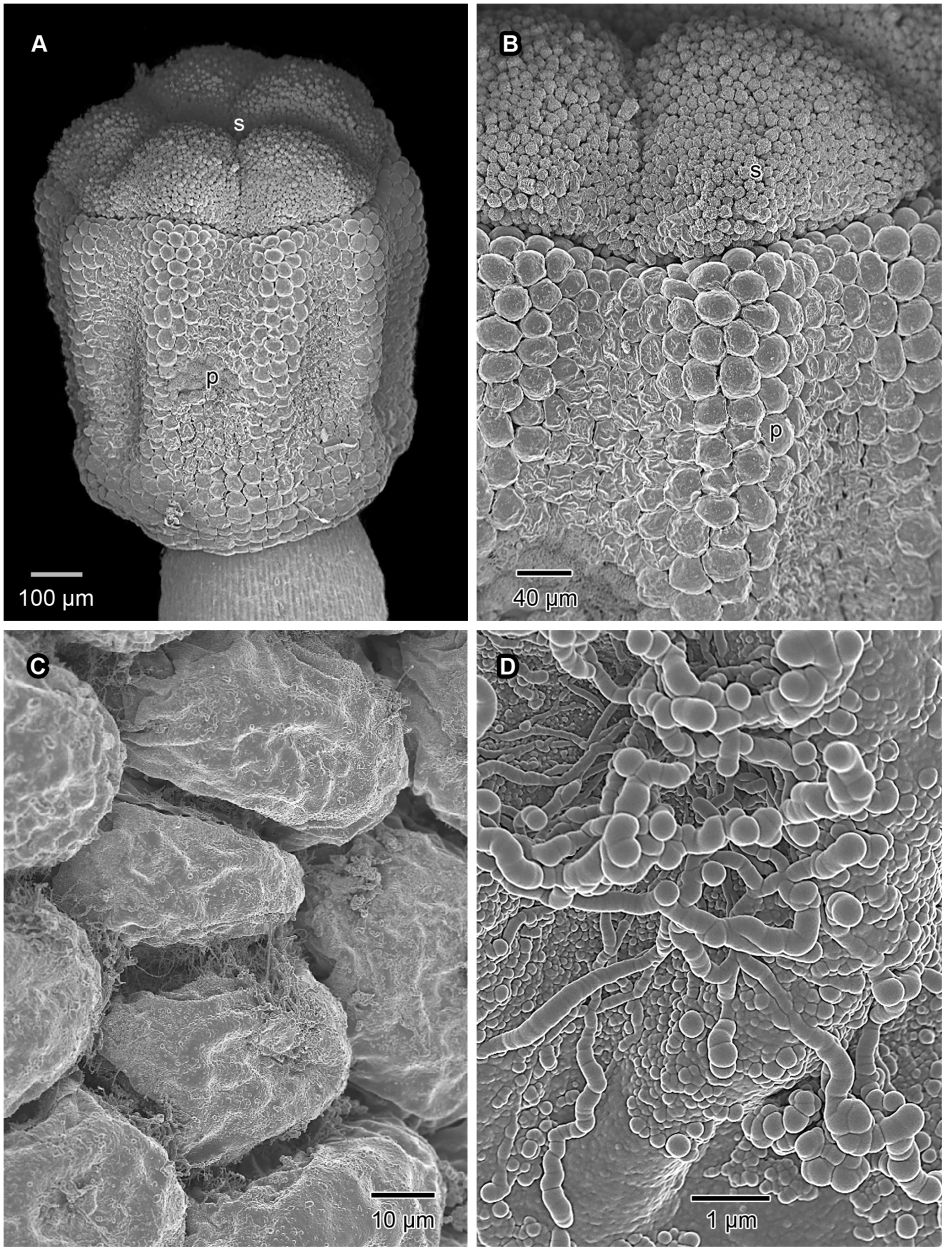
Micromorphology of stylar head complex. SEM micrographs illustrating surface topographical features, both in freshly-fixed, critical point dried material (A & B), and in unprocessed samples from herbarium specimens (C & D). In Vanguerieae the stigma (s) terminates the pollen presenter (p), the whole being referred to as the “stylar head complex”. In freshly-fixed pollen presenters taken from buds just prior to anther dehiscence, as in the case of A and B, the outer surfaces are largely devoid of adherent material. (A) Stylar head complex from an almost mature flower bud showing the slightly longitudinally-grooved pollen presenter and the terminal stigmatic lobes. (B) Enlarged portion of the stylar head complex illustrating the sharp boundary between the pollen presenter with its relatively large, convex, outer tangential epidermal cell walls, and the stigmatic lobes with their much smaller, rather papillate epidermal cells. (C) Surface of pollen presenter from an open flower showing secretions (initially somewhat sticky), especially in sinuses between epidermal cells. (D) Enlarged surface of pollen presenter showing secretions apparently exuded from the epidermal cells; these appear globular, or thread-like and often somewhat moniliform.

**Figure 3:**
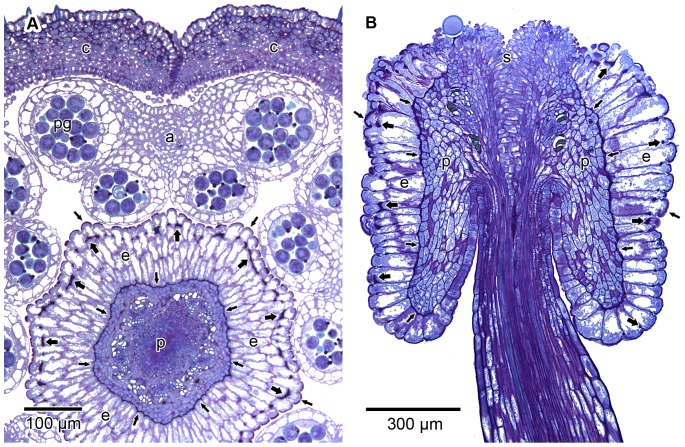
Anatomy of stylar head complex. LM transverse and longitudinal sections illustrating the anatomy of the pollen presenter (p) and associated structures; GMA-embedded, stained with periodic acid Schiff/toluidine blue. (A) Segment of a transverse section through a flower bud showing position of corolla lobes (c) and a still intact anther (a) with four pollen-filled microsporangia, two of the latter appressed to, and fitting into the longitudinal grooves of, the centrally placed pollen presenter (p). Note ring of well-developed vascular tissue (vessels without stained contents) in central parts of the presenter. Epidermal cells (e) of the pollen presenter are radially elongated (appearing multiseriate due to slightly oblique angle of section). Note darkly stained cell wall thickenings of Igersheim, some indicated with thin (on outer and inner periclinal cell walls) or thick (bands encircling anticlinal cell walls) arrows. Pollen grains (pg) have similar darkly stained protruding onci (compare Figure 12). (B) Stylar head complex in longitudinal section to show the pollen presenter with recessed style. Note prominently enlarged and radially elongated epidermal cells, as well as the terminally placed stigma (s), the latter with a single adherent pollen grain; labels as in A.

In *V. infausta*, secondary pollen presentation follows the known pattern for the Vanguerieae (described in Introduction above; Figure 1). In open flowers, the style is 4.5–6 mm long and ends in a stylar head complex into which the style is recessed (Figure 2; 3B). The stylar head complex is about 1 mm long and somewhat versatile. The pollen presenter is demarcated from the stigmatic surface by its larger epidermal cells (Figure 2A, B). The pollen is bright yellow and is deposited on the upper two thirds of the pollen presenter (Figure 1B), the surface of which is shallowly grooved longitudinally (Figure 2A, B). The ventral pollen sacs of each anther fit exactly in the grooves of the pollen presenter (Figure 3A). Adherence of the pollen is facilitated by sticky secretions on the surface of the pollen presenter (Figure 2C, D) and on the pollen grains themselves.

### Epidermis of the pollen presenter

**Figure 4:**
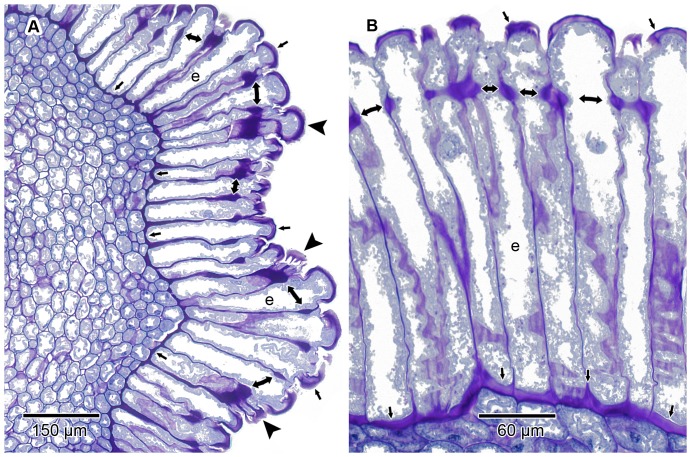
Anatomy of pollen presenter epidermis. LM transverse sections illustrating the anatomy of the pollen presenter epidermis (e); GMA-embedded, stained with periodic acid Schiff/toluidine blue. Wall thickenings of Igersheim are amorphous and stain dark purple-pink. Some of the thickenings are denoted by either single- (those of outer and inner tangential walls) or double-headed (bands of Igersheim) arrows. (A) Epidermis comprising of markedly radially-elongated cells and overlying the mesophyll with its much smaller and isodiametric parenchyma cells. Epidermal cells of longitudinal grooves are radially slightly shorter than those of the ridges. Three arrowheads (without shafts) point to epidermal cells in which are visible traces of one or more radial pectinaceous strands linking the band of Igersheim and the outer tangential cell wall thickening (most obvious in lowermost cell). These difficult-to-see radial strands are visible in sections of anticlinal walls of epidermal cells cut parallel with the surface. (B) Epidermal cells showing the wall thickenings of Igersheim. Thickenings of inner tangential walls often extend distally for a short distance along the anticlinal walls.

**Figure 5:**
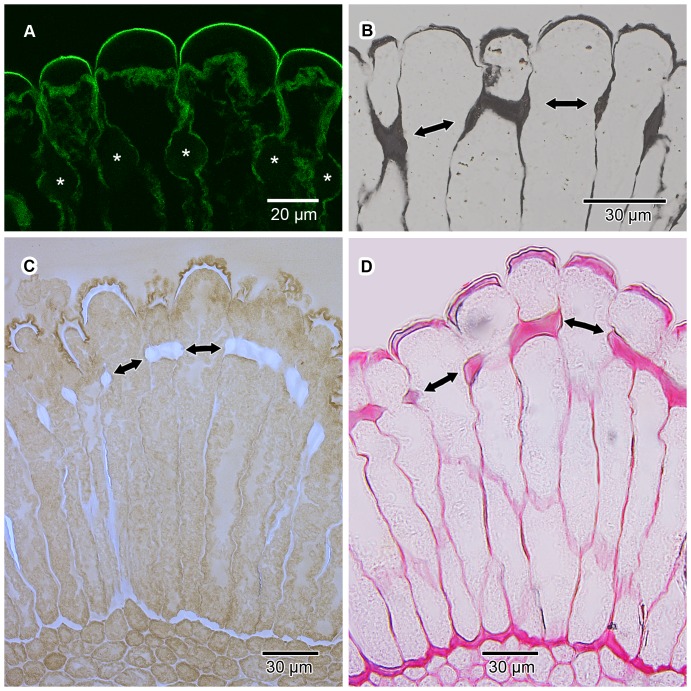
Histochemical staining of epidermal cell walls. Transverse sections of GMA-embedded pollen presenters treated with various histochemical stains to establish the composition of the wall thickenings of Igersheim in the epidermis; double-headed arrows indicate position of bands of Igersheim. (A) Auramine O under CLSM; this stain is a fluorescence enhancer with a strong affinity for regions containing acidic and unsaturated waxes, as well as cutin precursors and suberin. Relatively thin cuticle covering the outer anticlinal walls clearly visible because of much stronger autofluorescence. Bands of Igersheim (asterisks) show no reaction, indicating absence of cutin and suberin. (B) Tannic acid-ferric chloride under LM. Black colour of walls and thickenings indicates the presence of calcium pectate. (C) IKI under LM; starch grains stain blue-black (not illustrated). Unexpectedly, the wall thickenings of Igersheim stain prominently pale blue, most probably indicating the presence of xyloglucan. (D) Ruthenium red under LM. Cell walls, and in particular wall thickenings, stain bright red, thus confirming the presence of de-esterified pectin. Mesophyll cell walls adjacent to the epidermal cells also stain intensely.

In *V. infausta* the epidermal cells of that part of the pollen presenter which carries the pollen are markedly radially elongated from an early stage of development. This is in agreement with the known anatomy of the stylar head complex in other members of the Vanguerieae (e.g. [4], [5], [22]). The consistent presence of the encircling cell wall thickenings towards the distal end of the epidermal cells previously reported by Igersheim [4], was confirmed (Figure 3; 4; 5). Like in other Vanguerieae [4], these thickenings become conspicuous when the epidermal cells are about twice as long as wide; they remain in their original position and are at the same level in all the cells when viewed in transverse section (Figure 3; 4). In addition, for the first time cell wall thickenings were recorded towards the proximal end of the epidermal cells. These thickenings are of a more irregular arrangement and development than the other thickenings. They mainly affect the inner tangential cell walls adjacent to subepidermal parenchyma cells (Figure 3; 4; 5). The distal (outer tangential) cell walls, below the cuticle, are also thickened (Figure 3; 4; 5) and connected by a few radial pectinaceous strands per cell to the encircling anticlinal wall thickenings. The radial strands (Figure 4A) can only be seen in rare sections of anticlinal walls of epidermal cells cut parallel with the surface. Igersheim [4] referred to the distal cell wall thickenings as the “cutinized outer tangential walls” of the epidermal cells, an interpretation not supported by our observations. As will be reported below, these, and all the other cell wall thickenings, are mainly pectinaceous in composition, with no evidence of cutinization. We propose “thickenings of Igersheim” as a general term to describe all the prominent pectinaceous thickenings of the primary cell wall associated with the epidermal cell walls of the secondary pollen presenter in the Vanguerieae. The more specific term “bands of Igersheim” is proposed for the prominent anticlinal wall thickenings invariably encircling each epidermal cell towards its distal end, just below the sinuses separating adjacent cells (Figure 3; 4; 5). This terminology honours Anton Igersheim (1954–) of the Natural History Museum Vienna, who first drew attention to these wall structures [4].

**Figure 6:**
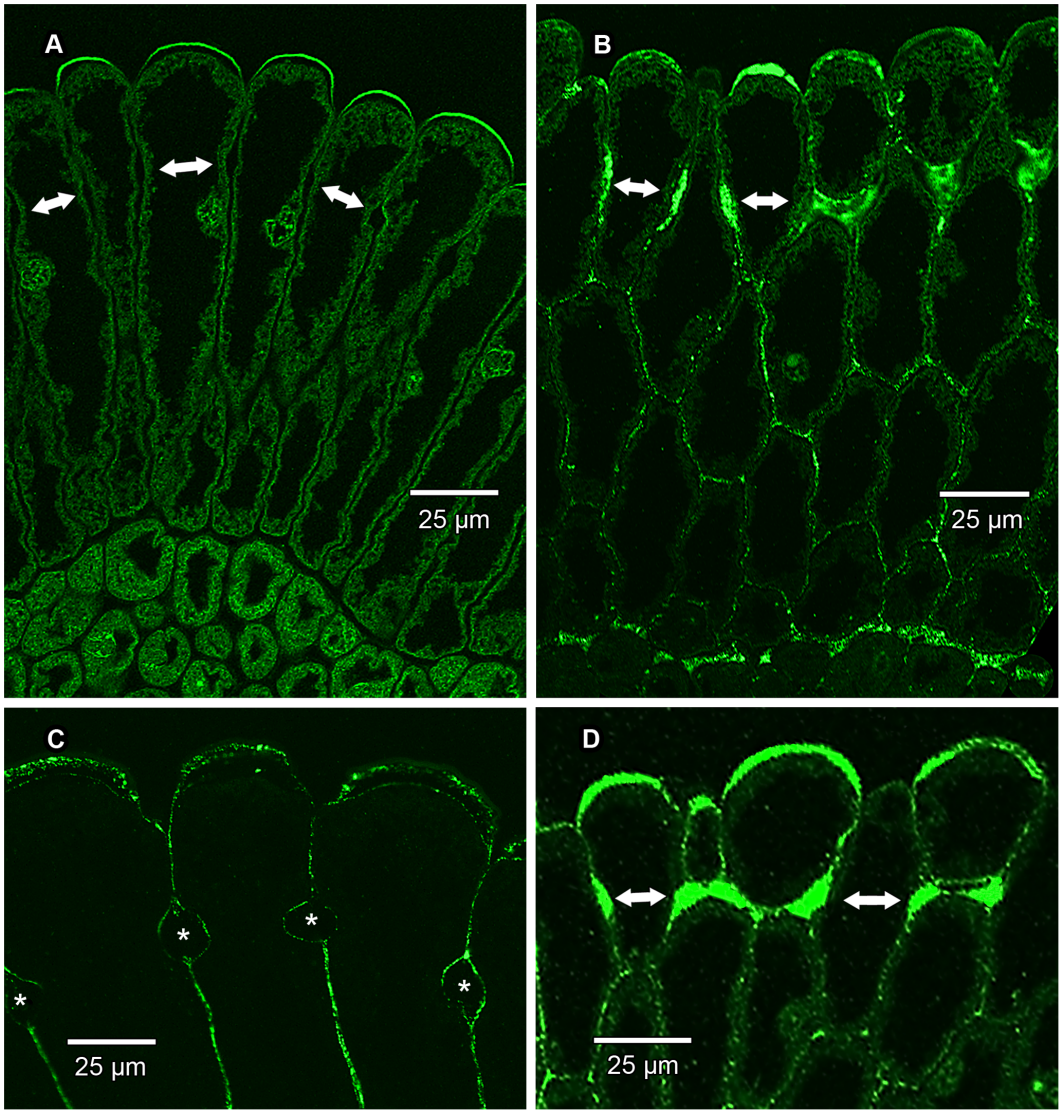
Immunolocalization of pectic homogalacturonan (HG) epitopes in epidermal cell walls. CLSM images of transverse sections of the pollen presenter epidermis. Double-headed arrows indicate position of bands of Igersheim. (A) Wax-embedded material immunolabelled with LM19 for de-esterified HG. This form of HG is mainly confined to those thickenings of Igersheim present in the outer tangential cell walls of the epidermal cells (for better resolution see C). Fluorescence of protoplasm in mesophyll cells due mainly to autofluorescence of chloroplasts. (B) Wax-embedded material immunolabelled with LM20 for methyl-esterified HG. This form of HG is abundantly present in primary cell walls as well as all the wall thickenings of Igersheim. (C) GMA-embedded material immunolabelled with LM19 and showing enlarged portion of distal end of epidermal cells. De-esterified HG mainly confined to outer portion (derived from primary cell wall?) of those thickenings of Igersheim present in the outer tangential cell walls of the epidermal cells, but also present in primary cell walls (middle lamella?) and outer boundary of bands of Igersheim (or electron-transparent zone between plasmalemma and wall thickenings—compare Figure 8), but essentially lacking in most of the bands themselves (bands marked with asterisks). (D) Wax-embedded material immunolabelled with LM20 and showing enlarged portion of distal end of epidermal cells. Methyl-esterified HG abundantly present in the bands of Igersheim, as well as throughout thickenings of the outer tangential cell walls.

The epidermis of the pollen presenter is covered by a relatively thin cuticle which was readily visible after staining with auramine O (Figure 5A). The cell walls, and in particular the thickenings of Igersheim, stained red using ruthenium red (Figure 5D). Similarly, the cell walls and their thickenings showed a positive reaction using the tannic acid-ferric chloride histochemical test (Figure 5B). These indicated the presence of de-esterified pectins and calcium pectate, respectively, in the cell walls. Positive staining (pink to dark purple) with the PAS reaction and toluidine blue supports a pectic composition for the thickenings of Igersheim (Figure 3; 4). All the histochemical tests for pectins were positive for these structures. Tagging with the anti-pectin monoclonal antibody LM20 indicated the abundant presence of pectic substances in these thickenings. LM19, which binds strongly to de-esterified pectic homogalacturonan, responds mainly to the outer (sub-cuticular) zone of the outer tangential epidermal cell wall thickenings and to a lesser extent to the middle lamella and the electron transparent zone between the plasmalemma and the cell wall thickenings of Igersheim (Figure 6A, C). LM20, indicative of esterified pectic homogalacturonan, strongly responds to the bands of Igersheim, as well as all of the thickenings along the outer and inner tangential cell walls (Figure 6B, D). IKI staining for starch grains was, rather unexpectedly, found to give an unusual pale blue (almost fluorescent) colour to all of the thickenings of Igersheim (Figure 5C). The significance of this response is not yet clear. Starch can be separated into two fractions, amylose and amylopectin. When performing the well-known IKI test for starch, it is the amylose that is responsible for the formation of the deep blue colour in the presence of iodine [23]. However, less well known is the fact that iodine in IKI also reacts with xyloglucan (“amyloid”) to give a deep blue colour [24]–[26]. Xyloglucan consists of a main cellulose chain with xylose or galacto-xylose side chains and is water-soluble, unlike structurally related cellulose, which is insoluble [27]. Xyloglucan is commonly associated with pectin [28] and together these two polymers may comprise approximately a third each of the polysaccharides of primary cell walls in dicotyledons [29]. Besides its role as a structural component in plant cell walls [30], xyloglucan also functions as a storage polysaccharide, especially in seeds [31]–[33]). We provisionally interpret the pale blue staining of the pectinaceous thickenings of Igersheim with IKI as indicative of the presence of xyloglucan.

**Figure 7:**
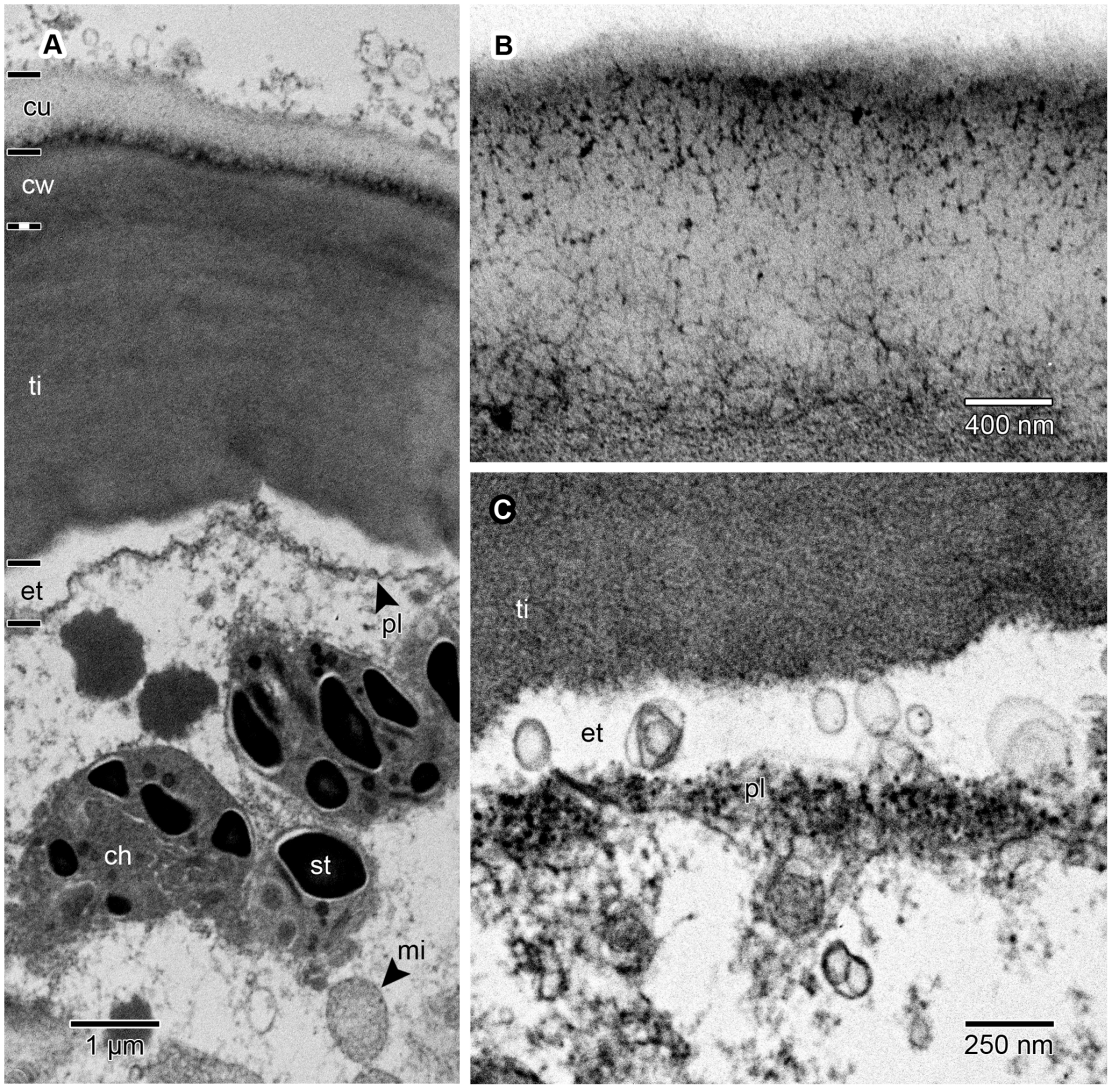
Ultrastructure of outer tangential cell wall thickenings and adjacent cuticle and protoplast of epidermal cells. TEM images of material postfixed in osmium tetroxide, embedded in SPI 812, and viewed in transverse section. (A) Outer tangential cell wall showing cuticle (cu) and slightly different electron-dense layers demarcated as the remains of the original primary cell wall (cw), thickening of Igersheim (ti), and an electron-transparent zone (et) (enlarged in C) between the wall and the plasmalemma (pl). The boundary between the primary wall and the thickening of Igersheim is not clear because the two layers merge gradually. Note extracellular deposits, probably including sticky subtances, on the cuticle. Visible in the cytoplasm are, among others, mitochondria (mi) and chloroplasts (ch) with starch grains (st). (B) Cuticle pervaded by a branched system of electron-dense fibrillar material, most probably representing microchannels. (C) Electron-transparent layer (compare A) between the plasmalemma and thickening of Igersheim showing several secretory vesicles. These vesicles are often seen to be fused to the plasmalemma and are evidently derived from the cytoplasm through exocytosis. It is suggested that these vesicles contain materials used in the formation of the cell wall thickenings; most probably also sticky secretions (compare Figure 2C, D) exuded from the cuticle surface. In young epidermal cells, and before the formation of wall thickenings (not illustrated), the cytoplasm next to the plasmalemma contains an abundance of rough endoplasmic reticulum; some of the ribosomes are still visible in the image; labels as in A.

**Figure 8:**
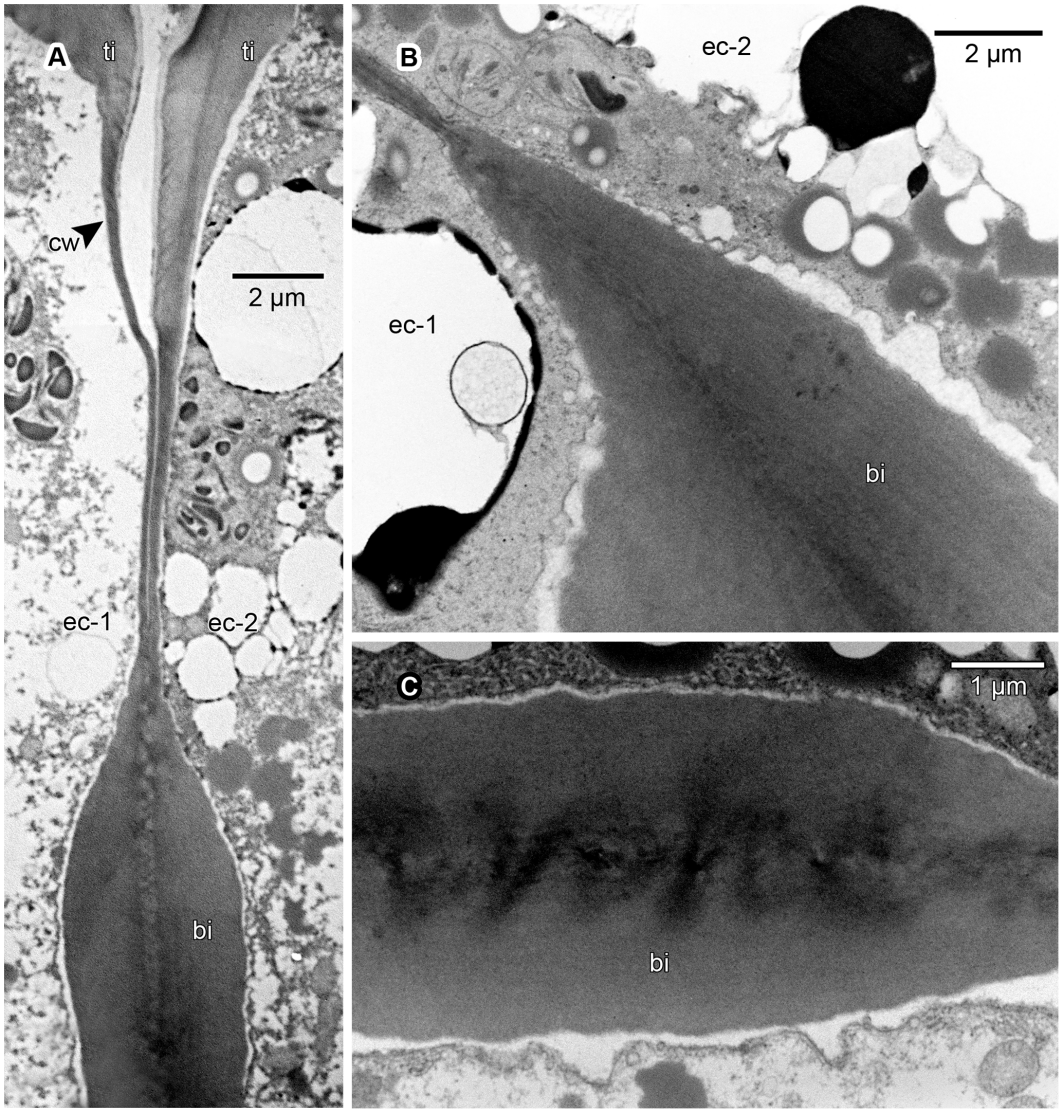
Ultrastructure of bands of Igersheim. TEM images of material postfixed in osmium tetroxide, embedded in SPI 812, and viewed in transverse section. Shown are the distal portion of two adjacent epidermal cells (ec-1 and ec-2), primary cell walls (cw), thickenings of Igersheim (ti) affecting tangential cell walls, and bands of Igersheim (bi). (A) Distal portion of two bordering epidermal cells showing a common band of Igersheim in transverse section. These bands develop in areas of primary wall rich in plasmodesmata, the remains of which persist as electron-dense transverse areas towards the centre (compare C). Transverse section through the distal portion of a band of Igersheim between two epidermal cells. Note faintly longitudinally-layered fibrillar structure of matrix and surrounding electron-transparent zone (compare Figure 7) between plasmalemma and band; labels as in A. (C) Enlarged central portion of a band of Igersheim (bi) between two epidermal cells showing electron-dense transverse zones reflecting the prior position of plasmodesmata in the common primary cell wall.

**Figure 9:**
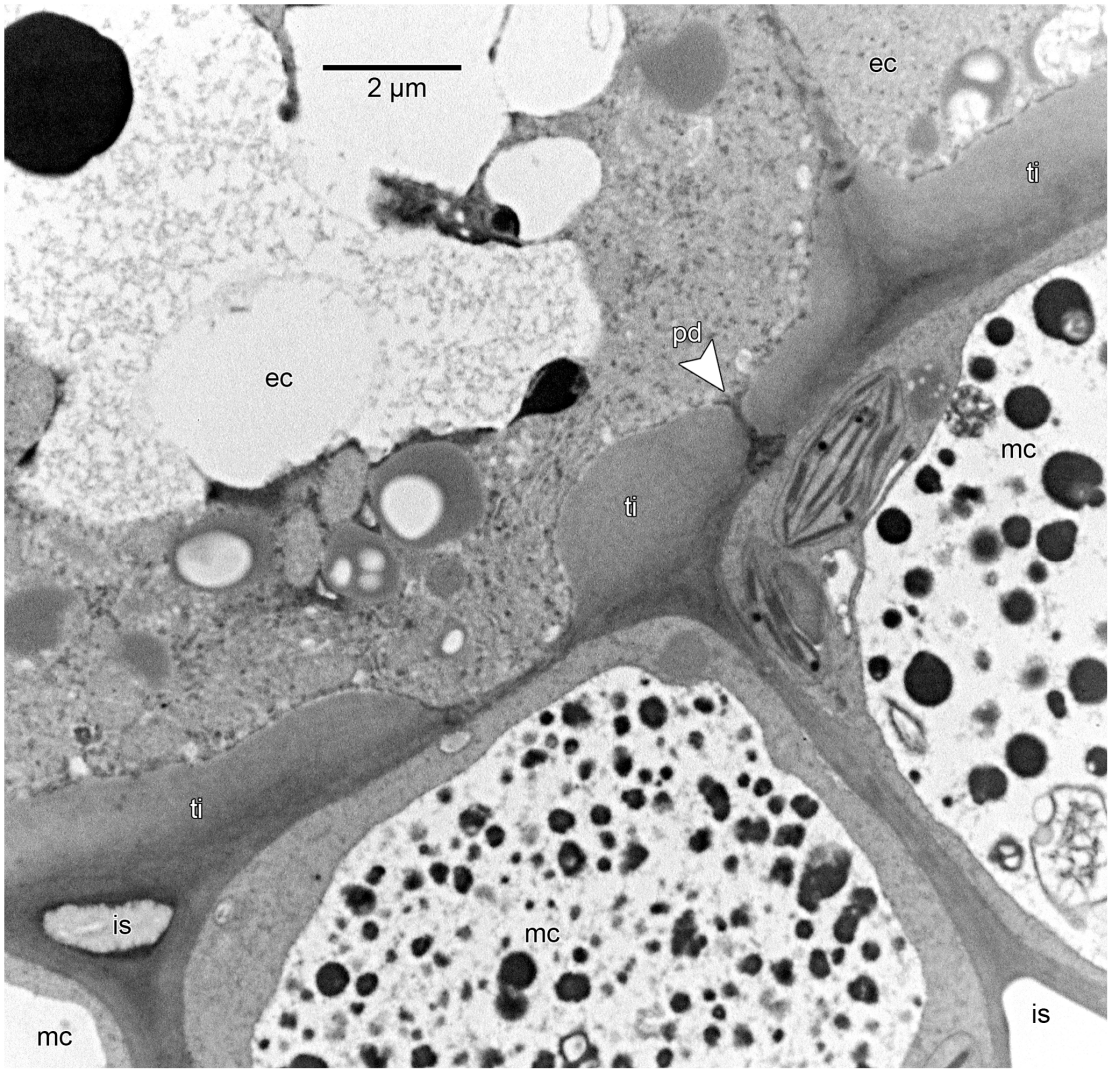
Ultrastructure of contact zone between epidermis and mesophyll. TEM image of material postfixed in osmium tetroxide, embedded in SPI 812, and viewed in transverse section. Shown are the proximal ends of two epidermal cells (ec) bordering three mesophyll cells (mc) and associated intercellular spaces (is). Thickenings of Igersheim (ti) are restricted to the walls of the inner tangential walls of the epidermal cells; the thickenings may affect only certain parts of the wall. Note single plasmodesma (pd) (compare Figure 10) in the thickening of Igersheim. Vacuoles of mesophyll cells filled with numerous osmiophilic droplets.

**Figure 10:**
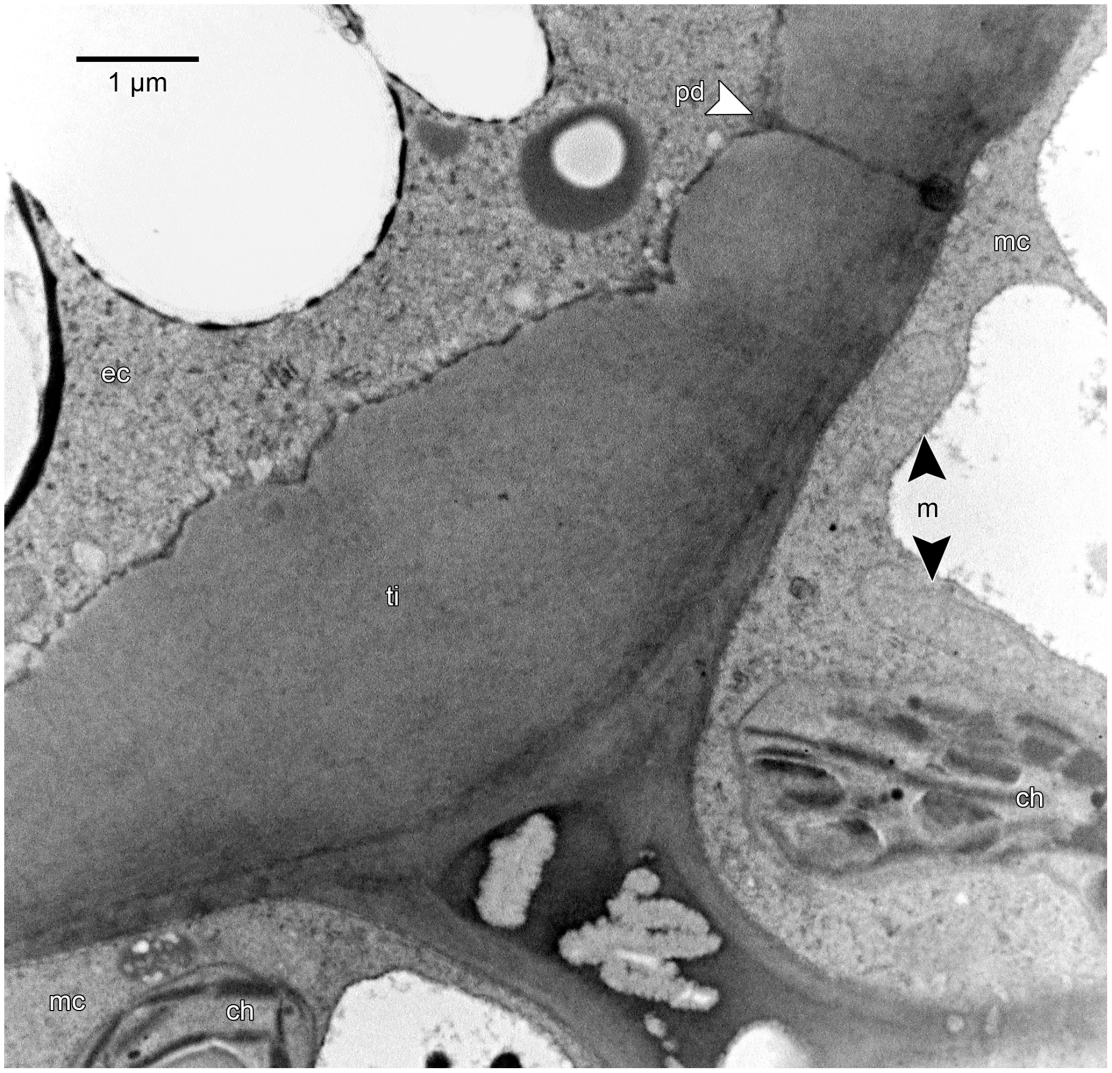
Ultrastructure of inner tangential wall thickenings of epidermal cells. TEM image of material postfixed in osmium tetroxide, embedded in SPI 812, and viewed in transverse section. Enlarged thickened inner tangential cell wall of an epidermal cell (ec) bordering two mesophyll cells (mc). Position of primary cell wall is still visible as a slightly more electron-dense layer to the outside of the thickening of Igersheim (ti). Note distinct plasmodesma (pd) traversing the thickening. Such well-defined plasmodesmata have not been observed in the bands of Igersheim (compare Figure 8) and seem to be confined to thickenings in the inner tangential walls of the epidermal cells. The mesophyll cells are rich in chloroplasts (ch) and mitochondria (m; rather faintly stained in this image).

**Figure 11:**
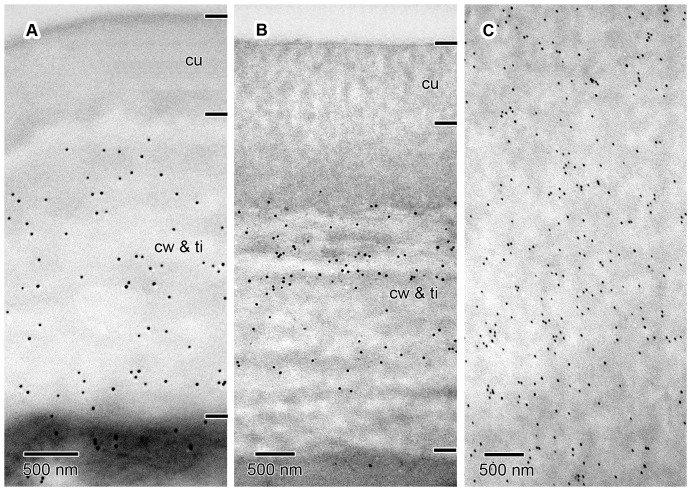
Immunogold labelling of pectic homogalacturonan (HG) epitopes in epidermal cell wall thickenings. TEM image of material embedded in LR White and examined unstained in transverse section. Short transverse lines along right-hand edge of images demarcate approximate boundaries between the cuticle (cu) and the cell wall thickening comprised of the primary cell wall (cw) and thickening of Igersheim (ti), the latter two layers intergrade and cannot be distinguished, especially in these unstained sections. (A) Outer tangential cell wall of epidermal cell immunolabelled with LM19 for de-esterified HG. This form of HG is present in most parts of the wall, but absent from the cuticle. (B) Outer tangential cell wall of epidermal cell immunolabelled with LM20 for methyl-esterified HG. This epitope is likewise absent from the cuticle, but mainly confined to the central portion of the wall thickening in this particular section. However, this distribution pattern is very variable and in other sections the methyl-esterified HG may be spread more or less throughout the wall thickening. (C) Enlarged portion of a band of Igersheim immunolabelled with LM20 for methyl-esterified HG. This epitope is abundantly and uniformly present throughout the thickening. This contrasts with de-esterified HG which is very sparsely present in these bands (not illustrated, but confirming pattern depicted in Figure 6A & C above).

Under TEM the internal structure of the thickenings/bands of Igersheim is usually faintly fibrillar and layered (Figure 7; 8; 9; 10). In this respect the ultrastructure of the thickenings is reminiscent of mucilaginous epidermal cell walls [34]. Immunogold reactivity supports the pectin composition of the thickenings (Figure 11). Initially plasmodesmata are very noticeable in those portions of the primary cell wall where the bands of Igersheim are initiated. As the bands develop, the plasmodesmata become less defined but their original presence is still apparent as diffuse, electron-dense bands in the central portion of the thickenings (Figure 8). Well-defined plasmodesmata were never observed in the fully developed bands of Igersheim. On the other hand, very distinct, though rather few plasmodesmata traverse the thickenings of the inner tangential epidermal cell walls (Figure 9). Such plasmodesmata are visible as narrow channels in the thickened part, from where they broaden out in the common primary cell wall between the epidermal and mesophyll cells (Figure 10). Membrane-bound vesicles or their membranous remnants were particularly noticeable next to wall thickenings, either in the cytoplasm along the plasmalemma, or in the electron-transparent zone between the plasmalemma and cell wall (Figure 7C; 8; 9; 10). We think they are exocytic vesicles contributing to the thickening of the cell wall. In the case of the outer tangential epidermal walls the vesicles could also be bringing a secretion for release to the outside of the cell.

**Figure 12:**
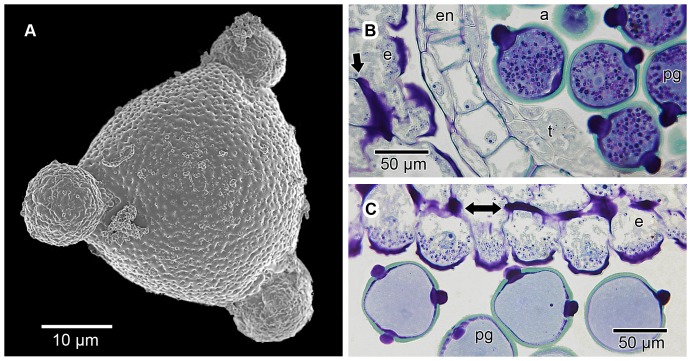
Morphology and contents of mature pollen grains. Mature pollen grains are suboblate to spheroidal, isopolar, radially symmetrical and 3-porate. A prominant globular protruding oncus projects from each aperture. For most of their development, the pollen grains do not contain starch grains. However, before being shed from the anthers, large numbers of starch grains become visible inside the pollen grains, as well as in the cytoplasm towards the distal end of the epidermal cells of the pollen presenter. Following the shedding of the pollen grains, most of the starch rapidly disappears from the grains, subsequently also from most of the epidermal cells. (A) SEM micrograph of a mature pollen grain after being shed from the anther, showing the three protruding onci. The tectum is perforate with a tendency towards microreticulate, but in unacetolyzed grains exine sculpturing is usually concealed by a layer of secretions. (B) LM image of a transverse section of pollen grains still in the anther, but shortly before being shed; GMA-embedded, stained with periodic acid Schiff/toluidine blue. At this stage the pollen grains contain abundant starch grains (stained dark pink-purple to black). The pollen grains have prominent protruding onci (stained dark purple-black) and are surrounded by the remains of the tapetum (t) on the inside, followed by the endothecium (en) and then the epidermis of the microsporangium on the outside. The anther is shown in close proximity to the epidermal cells (e) of the pollen presenter, the latter also with minute starch grains concentrated in the cytoplasm below the outer tangential cell walls of the epidermal cells. The arrow denotes a band of Igersheim. (C) LM image of a transverse section of pollen grains with protruding onci shortly after having been shed from the anther; GMA-embedded, stained with periodic acid Schiff/toluidine blue. Also shown are the associated epidermal cells (e) of the pollen presenter. At this stage most of the starch grains have disappeared from the pollen grains. However, minute starch grains are still abundantly present in the cytoplasm of the epidermal cells, concentrated between the distally located bands of Igersheim (double-headed arrow) and the outer tangential cell wall.

In flower buds and shortly after anthesis, the epidermal cells of the pollen presenter (Figure 3; 4) which face the anthers have an intricate ultrastructure with an abundance of organelles, including smooth and rough endoplasmic reticulum, Golgi apparatus, mitochondria and secretory vesicles (Figure 7A, C). This suggests considerable metabolic activity indicative of an active functional role for the epidermal cells. Shortly before and at the time of pollen release by the anthers, starch grains (confirmed with IKI) were abundant in the epidermal tissue of the pollen presenter where they were concentrated distally (Figure 12B, C). Besides in these epidermal cells (Figure 7A), many cells to the interior (mesophyll) also contain several chloroplasts and contribute to the pale greenish colour of the pollen presenter (Figure 1; 10).

In transverse sections the pollen presenter is seen to have well-developed vascular tissue (Figure 3A). This, as well as the presence of conspicuous pectinaceous thickenings, suggest a potential rehydration role for the pollen presenter. Moreover, what appears to be a copious secretion is visible on the outside of some of its epidermal cells (Figure 2C, D; 7A). Ultrastructurally the cuticle is pervaded by a branched system of fibrillar material, most probably representing microchannels (Figure 7B) capable of material transport. An abundance of dictyosomes (Golgi apparatus) and membrane-bound vesicles in the cytoplasm of the epidermal cells suggests a potential secretory function for these cells. Yet another hypothesis is the secretion, through exocytosis, of substances either to facilitate coherence of the pollen grains around the pollen presenter (in addition to sticky material possibly derived from the tapetum) or for uptake by the grains themselves for a specific function. In herbarium specimens of *V. infausta*, as in other Vanguerieae (unpublished observations) the pollen grains seem to be “glued” together as well as “glued” to the pollen presenter. With SEM, threads are sometimes visible between pollen grains and the surface of the pollen presenter (Figure 2C, D). We consider these threads to be sticky material. However, at present, the relative contribution of the pollen presenter and the anthers (tapetum and/or pollen grains) towards the secretion of sticky substances is unclear; both sources are likely to be involved. All of these observations suggest support for both a secretory and hydrating function for the pollen presenter.

### Pollen grains and protruding onci

In *V. infausta* the pollen grains are shed as monads (Figure 12). They are suboblate to spheroidal, isopolar, radially symmetrical, about 70 µm in diameter (somewhat smaller when dried) and 3-porate. A prominent globular protruding oncus projects from each aperture. The tectum is essentially perforate with a tendency towards microreticulate, but in unacetolyzed grains sculpturing is usually concealed by a layer of secretions.

**Figure 13:**
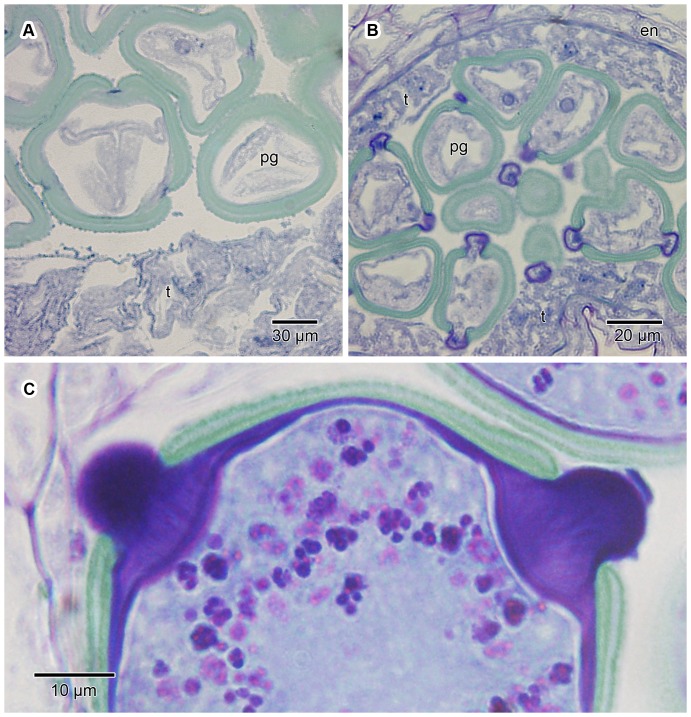
Development of the protruding onci in pollen grains. LM transverse sections of anthers showing two stages of immature pollen grains (pg), and a mature grain, in the microsporangium; GMA-embedded, stained with periodic acid Schiff/toluidine blue. The protruding onci only make their appearance at a fairly advanced stage of pollen grain development. The microsporangium is lined with a prominent, multilayered, partly amoeboid tapetum (t). At least the inner cells of the tapetum degenerate as the pollen approaches maturity and are suspected to deposit, amongst others, sticky secretions on the grains. (A) Young pollen grains having reached more or less mature size, but still without protruding onci. A small, dark blue spot below each aperture, as well as an associated thickening of the intine (oncus), indicates the point from which the protruding oncus is about to develop. A deposit (stained pale bluish) is visible on the outer surface of the grains. (B) Later developmental stage than in A, showing the first appearance of the protruding onci. Initially the walls of the protruding onci stain less intensely than in mature grains (compare C). (C) Mature pollen grain just before it is shed, showing two protruding onci and starch grains. The intine lining the exine and the protruding onci is an essentially continuous layer.

In young flower buds, the microspores do not display protruding onci till well past the tetrad stage (Figure 13A). However, before dehiscence of the anthers, a conspicuous oncus (thickening of the intine) becomes evident below each aperture (Figure 13B). This oncus is continuous with material subsequently extending through the aperture forming a protruding oncus. The thickenings of Igersheim in the epidermal cells are not visible in the very young epidermis but develop before protruding onci appear.

**Figure 14:**
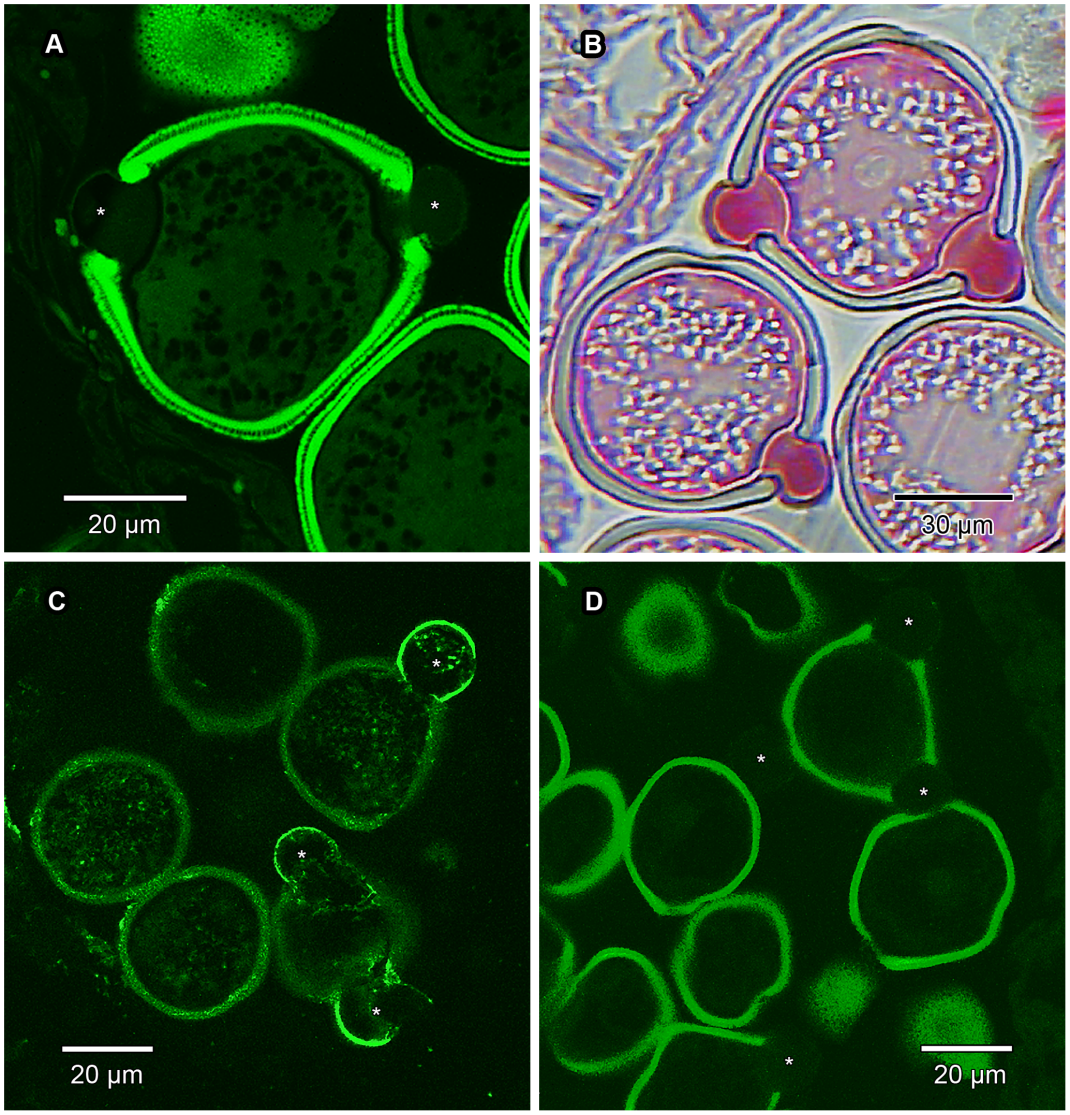
Histochemical staining and immunohistochemistry of pollen grains. Transverse sections of GMA- or wax-embedded pollen grains treated with histochemical stains and fluorescent monoclonal antibodies to elucidate the composition of the protruding onci (marked in some images with an asterisk). (A) Auramine O in GMA-embedded material under CLSM. Exine of pollen grains show enhanced autofluorescence, but the protruding onci show no reaction, indicating absence of cutin and suberin. (B) Ruthenium red in GMA-embedded material under LM. Protruding onci stain bright red, thus confirming the presence of pectin. (C) Wax-embedded material immunolabelled with LM19 for de-esterified pectic homogalacturonan (HG) and viewed under CLSM. This form of HG is mainly confined to the outer layers of the protruding onci, and possibly also (though sparsely so) in the exine and intine. Exine visibility enhanced due to it being autofluorescent. (D) Wax-embedded material immunolabelled with LM20 for methyl-esterified HG and viewed under CLSM. This form of HG is apparently absent from the protruding onci, and seemingly also the rest of the pollen grain. Exine clearly visible due to autofluorescence.

Pollen grains treated with auramine O did not show any fluorescent enhancement of the onci, indicating the absence of cutin and suberin (Figure 14A). The outer portion of the protruding oncus stained intensely with ruthenium red (Figure 14B) as well as with the PAS/toluidine blue reaction (Figure 3A; 12B, C; 13B). Initially the PAS/toluidine blue-stained area is faint (Figure 13A), but shortly afterwards a clear outer purple layer is visible (Figure 13B). This indicates the abundant presence of pectic (or mucilaginous as they are sometimes referred to) substances. LM19 for de-esterified pectic homogalacturonan also responded positively (Figure 14C) for the outer layer of the protruding portion of the oncus. LM20, on the other hand, indicated that esterified pectic homogalacturonan is apparently absent from the protruding onci, and seemingly also the rest of the pollen grain (Figure 14D).

The protruding oncus is derived from the intine and is essentially an inflated (thickened) pectin-impregnated part of the intine associated with an aperture (Figure 12B, C; 13C). Although we have not studied the ontogeny of the protruding onci of *V. infausta* at ultrastructural level, this was shown in other Rubiaceae to be an intricate process [9]. Kuang et al. [9] studied the main stages in the formation of protruding onci in *Uncaria hirsuta* using TEM. They describe the “final” protruding oncus before dehiscence of the anthers as having an outer electron-dense layer and an extremely thick electron-translucent core. This concurs with the present study where protruding onci are very intensely stained (both with the PAS/toluidine blue and ruthenium red reactions) indicating that the electron-translucent core may be composed of microfibrils interspersed with pectin. In transverse section the ultrastructure of this inflated part of the intine is not only reminiscent of mucilaginous cell walls [34], but in staining intensity and histochemistry also resembles the thickenings of Igersheim in the pollen presenter (Figure 3A; 12B, C; 13C). In transverse section, a developing oncus is seen to have an often weakly horizontally-layered central portion which stains bluish with the PAS/toluidine blue reaction (Figure 13B). Up to this stage the multilayered, partly amoeboid tapetum of the anther is conspicuous (Figure 13A, B), but subsequently, the tapetum becomes reduced. In the course of development, the protruding onci become more and more intensely stained and by the time the pollen grains are ready to be shed, the protruding onci are so deeply stained that it is difficult to detect any internal structure (Figure 12B, C; 13C). The intine lining the exine and the protruding onci is visible as an essentially continuous layer (Figure 13C).

After dehiscence of the anthers, the protruding onci appear slightly smaller and less deeply stained (compare Figure 12B, C). A faintly fibrillar content may be seen. The compact inner zone of the intine below the protruding oncus (Figure 13C) concurs with the description of the “inner microfibrillar cellulosic layer” of the oncus from which the pollen tube emerges in germinating pollen grains of *Corylus avellana* (Betulaceae) and *Eucalyptus rhodantha* (Myrtaceae) [10], [11]. In contrast to the observations of Kuang et al. [9], in *V. infausta* after anther anthesis, the protruding onci remain conspicuous, though somewhat shrunken. We suspect this apparent “shrinkage” of the protruding onci may be due to harmomegathic change [11], [35]–[37] reflecting the hydration status of the pollen grains.

Starch granules were not conspicuous in the pollen grains or other parts of young flower buds. However, shortly before anther dehiscence, at the stage of most intense staining of the intine (including of the protruding onci), a large number of purple granules (PAS/toluidine blue reaction) were visible in each pollen grain (Figure 12B; 13C). The IKI test for starch was positive for these granules. After anther dehiscence, they were essentially absent in the pollen grains but could still be seen in the epidermal cells of the secondary pollen presenter (Figure 12C). We hypothesise that this disappearance of the starch may well be linked to changes in the water potential (through an increase in sugar concentration) of the pollen grains.

### Possible functional association between pollen presenters and protruding onci

An initial suggestion was that the bands of Igersheim may act as mechanical barriers preventing pollen tubes, from grains that may accidentally germinate on the pollen presenter, from penetrating its tissues [4]. However, in our observations on pollen presenters in Vanguerieae no signs of germinating grains were found. Earlier reports [22] of germinating grains are most probably based on an erroneous interpretation of protruding onci as pollen tubes, the true structure of the former not being known at the time. This “mechanical barrier” hypothesis may initially appear attractive considering a superficial similarity in form and arrangement between the bands of Igersheim in the pollen presenter and the Casparian strips (bands of Caspary) associated mainly with the endodermal cells in the root. In both cases the bands are on anticlinal walls perpendicular to the surface of their respective organs. However, Casparian strips are chemically and functionally quite different [38]. The latter is impregnated with mainly lignin, most likely also some other hydrophobic substance(s) which may sometimes include suberin [38]. The bands are hydrophobic, thus precluding the passage of water and solutes across these walls [38], [39]. This contrasts with the bands of Igersheim which are impregnated mainly with pectin, a group of complex polysaccharides known to be hydrophilic, thus most likely facilitating the passage of water and solutes across these walls—this by analogy to one of the roles ascribed to the pectin-rich internal onci in pollen grains of other plants [10]–[12]. Xyloglucan, apparently also present in these thickenings, likewise strongly retains water [40]. Moreover, it is now well established that pectic polysaccharides are not merely a jelly-like matrix found predominantly in the middle lamella, but substances of considerable chemical complexity, comprising a substantial, and orderly deposited, component of the cellulose-hemicellulose network in the cell wall [41]–[45].

Pollination in flowering plants is one of a sequence of relatively rapid and ephemeral events in floral biology. Usually, the entire pollination process requires only a few hours, and frequently occurs over a period of two days or less [35]. It is generally accepted that all pollen grains undergo pronounced harmomegathic change as a result of partial desiccation following exposure to air [37]. However, to maintain its ability to germinate rapidly, the pollen protoplast must remain sufficiently hydrated to remain physiologically active and responsive. For most flowering plants, and especially those reliant on outcrossing, a major obstacle must be the prevention of excessive protoplast desiccation while undergoing transfer from the newly dehisced anther to a receptive stigma [35]. The risk of dehydration for the pollen grains must therefore be particularly high in those flowering plants with secondary pollen presentation, unless special mechanisms operate to limit desiccation. In the Vanguerieae, as in most other taxa with secondary pollen presentation, the mass of pollen is transferred from the protective anthers to the pollen presenter just prior to anthesis, after which it is carried by the style to usually well above or away from the other floral parts and in full exposure to the atmosphere (compare Figure 1B). The stress-tolerant (“robust”) pollen grains [46] of the genus *Eucalyptus* are from flowers where, after anthesis, the dehisced anthers remain fully exposed and without protection from heat and desiccation. It may be noteworthy that the pollen grains in this genus often have prominent internal onci [10].

Based on our anatomical observations, we suggest an active physiological role for the epidermis of the pollen presenter, rather than mere physical support for the adherent pollen grains as has hitherto been assumed. Here we put forward the hypothesis that the thickenings of Igersheim, as well as the protruding onci of the pollen grains, are functionally associated and part of the adaptive syndrome of secondary pollen presentation in the Vanguerieae. Investigation of the epidermal cell walls of pollen presenters of other Rubiaceae or angiosperm taxa may reveal that our hypothesis can be generalised. The hydrophilous nature of pectins and their abundant presence in the thickenings of Igersheim, as well as other cell walls of the pollen presenter and the protruding onci, suggest a possible apoplastic pathway for the movement of water and solutes from the vascular tissue of the pollen presenter towards the epidermis, and from there to the adherent pollen grains assisted by the connections provided by the protruding onci. In addition, the passage of sticky and possibly other secretions between the epidermal cells and the pollen grains may be facilitated by the thickenings of Igersheim and the pathways provided by the protruding onci. A cursory survey of the literature shows that pollen grains with protruding onci (or at least prominent intine projections from the apertures) are, in addition to various Rubiaceae other than Vanguerieae [2], frequently found in those members of the Proteaceae with secondary pollen presentation [47]–[50]). This may well support the suggested functional relationship between protruding onci and certain forms of pollen presenters. Furthermore, modified epidermal cells located on the pollen presenter adjacent to the anthers are not confined to the Rubiaceae, though within the family radially elongated cells may well be exclusively associated with the Vanguerieae [4]. For example, in the Proteaceae, *Banksia tricuspis* has “inflated” epidermal cells [51] [and in figure 7, p. 453, traces of possible cell wall thickenings near the distal end are visible] and in *Dryandra praemorsa* the ribs of the pollen presenter are formed by “inflated” epidermal cells. Similarly *Banksia ericifolia* has “enlarged polyphenol-containing” epidermal cells [52] with traces of possible cell wall thickenings evident towards the distal end in the figure (figure 13, p. 390). The co-occurrence of protruding onci and modified epidermal cells sometimes with pectic wall thickenings, an unusual combination, points to a possible functional association which seemingly deserves further study.

In the secondary pollen presentation system of the Vanguerieae, the receptive stigmatic surface is initially often (though not in *V. infausta*) concealed between appressed stigmatic lobes [4]. The epidermal cells of the pollen presenter may also function in the opening and closing of the stigmatic lobes in response to its different phases of receptivity. Ultrastructural similarities of the epidermal cells with the motor and associated cells in the pulvini of legumes [53] support such a suggested hydrostatic function.

## Conclusions

In flowers of *V. infausta* pollen is deposited by the anthers on a pollen presenter which is closely integrated with the stigma to form a structural unit known as the stylar head complex. Prominent cell wall thickenings associated with the epidermal cell walls of the secondary pollen presenter are composed mainly of pectin, with the possible addition of xyloglucan. Depending on position, we propose the terms “thickenings of Igersheim” and “bands of Igersheim” for these cell wall thickenings. The chemical composition of these thickenings does not support the hypothesis that in particular the bands of Igersheim may act as mechanical barriers preventing selfing by pollen tubes from grains that accidentally germinate whilst on the pollen presenter. We have also not noted any such germinating grains in *V. infausta*; an earlier claim for some Vanguerieae was most probably based on mistaking the protruding onci for pollen tubes.

The pollen grains of *V. infausta* have prominent protruding onci, the composition of which is also pectinaceous. Structurally the secondary pollen presenter is well supplied with vascular tissue and the epidermal cells are rich in organelles indicative of considerable metabolic activity including the secretion of substances. We hypothesise that the disappearance of starch granules from the pollen grains upon their release from the anthers may well be linked to changes in the water potential (through an increase in sugar concentration). As interface between the pollen presenter and the adherent pollen, we suggest an active physiological role for the epidermis of the pollen presenter, including the secretion of substances and the provision of water and other compounds to the pollen grains. We also suggest a functional link between the protruding onci and the wall thickenings of the epidermal cells of the pollen presenter.

Further work is required to test whether the proposed association between protruding onci and pectinaceous thickenings in the pollen presenter holds true for other members of the Rubiaceae with secondary pollen presentation, and even in members of other families of flowering plants that have pollen with prominent protruding onci. Assuming that active exchange of materials between pollen presenter and pollen grains is indeed facilitated by the thickenings of Igersheim and protruding onci, the detailed chemical composition of these thickenings and onci may even have practical implications beyond the mere understanding of their functional significance in the plant. One such field is the development of biodegradable polysaccharide microparticles used as delivery systems for drugs, as well as in other biomedical applications (e.g. [40], [54], [55]).
